# Editorial: Artificial intelligence applied to coronary artery diseases: from pathophysiology to precision medicine

**DOI:** 10.3389/fcvm.2023.1247620

**Published:** 2023-08-09

**Authors:** Ramona Vinci, Alessia d’Aiello, Sarah Costantino, Nikola Dino Capocchiano, Joan T. Matamalas, Daniela Pedicino

**Affiliations:** ^1^ Department of Cardiovascular and Pulmonary Sciences, Catholic University, School of Medicine, Rome, Italy; ^2^ Department of Cardiovascular Sciences, Policlinico Universitario Agostino Gemelli IRCCS, Rome, Italy; ^3^ Center for Translational and Experimental Cardiology (CTEC), Department of Cardiology, University Hospital Zurich, University of Zurich, Schlieren, Switzerland; ^4^ Real World Data Facility, Gemelli Generator, Fondazione Policlinico Universitario Agostino Gemelli IRCCS, Rome, Italy; ^5^ Cardiovascular Medicine, Center for Interdisciplinary Cardiovascular Sciences, Brigham and Women’s Hospital, Harvard Medical School, Boston, MA, United States

**Keywords:** plaque phenotyping, coronary artery disease, shear stress, immune system, inflammation, precision medicine, artificial intelligence

**Editorial on the Research Topic** Artificial intelligence applied to coronary artery diseases: from pathophysiology to precision medicine

The number of people who die each year from cardiac events is steadily increasing. Several unaddressed needs persist in the field of cardiac care, with coronary artery phenotyping and cardiac event stratification emerging as substantial challenges within the personalized medicine framework. Successfully tackling these issues could lead to significant reductions in routine diagnostic practices, allowing for more customized interventional strategies. Artificial intelligence (AI) and complex biological data could be an effective combination as novel preventive approaches applied to innovative cares. As a specialized branch of AI, machine learning (ML) allows deciphering composite clinical and biological conundrums, starting from big data, leading to the advanced identification of specific clinical variables and biological pathways. With this Editorial, we would like to collect the recently submitted experimental advances in management of patients presenting with coronary artery disease (CAD) assessed through some of the latest AI techniques.

In 2013, the concept of “pre-test probability” (PTP), centered on variables such as sex, age and clinical characteristics, was introduced, and then updated with the developing of a clinical likelihood ratio of CADs (from low to high) (1). Nevertheless, it has been proved that this model could overestimate the obstructive CAD prevalence, affecting patient management outcome. Is it possible to efficiently assess the probability of obstructive CADs working on relevant clinical data supported by AI algorithms? In their original investigation Kim et al. used a ML-based model, the CatBoost model (2), for predicting obstructive CAD. The authors used data derived from clinically relevant biomarkers further classified employing the SHapley Additive exPlanations (SHAP) post-hoc method, a game in which values act as players in a coalition with the goal of calculating each player's contribution to the match, thus releasing a prediction (3). According to SHAP explanation method, factors as troponin T, hemoglobin A1c, triglyceride, creatinine clearance, and high-density lipoprotein cholesterol were more predictive than diabetes mellitus, chronic kidney disease or dyslipidemia. Indeed, results demonstrated that their algorithms significantly improved the selection of patients presenting with stable obstructive CAD, when compared with classical PTP models. Such AI-based prediction models have the potential to nudge health systems towards more sustainable practices and streamline patient management.

Undoubtedly, AI tools can also be useful for rationalizing the healthcare costs, such as decrease the number of exercise electrocardiography, nuclear stress testing or stress echocardiography, and, although rare, their clinical adverse events. The IDENTIFY clinical trial by Stuckey et al. described a ML-based detection model, called CAD model, of functionally significant CAD in patients at rest who underwent left heart catheterization or coronary computed tomography angiography. The authors demonstrated that the use of variables stemmed from two non-invasive techniques, i.e., time synchronized orthogonal voltage gradient and photoplethysmographic signals, could be used as a first-line non-invasive diagnostic test. These methods could provide predictive insights, complementing traditional clinical tests and consequently leading to a reduction in subsequent costs. In their brief research report, Rousseau-Portalis et al. conducted a retrospective cross-sectional study applying an unsupervised learning and data clustering method for cardiovascular risk stratification. Using ML-algorithms, the authors showed, for the first time, that an important clinical feature, the carotid-femoral pulse wave velocity (PWT), was significantly correlated with presence and progression of coronary calcium score in participants at higher cardiovascular risk. From a pathophysiology point of view, clustering may promote better identification and subsequent classification of novel biological markers associated with specific patient subgroups, demonstrating, once more, the improvement of outcome prediction.

Cardiovascular imaging tools, such as echocardiography and cardiac magnetic resonance, represent one of the real-world applications, enabling an automated morphological outlining of cardiac elements, based on optimized-algorithms. In this regard, the observational study proposed by Diana et al. concluded how automatic myocardial strain imaging tool alongside a circulating biomarker, such as N-Terminal Pro-B-Type Natriuretic Peptide (NT-proBNP), could provide relevant clinical insights, identifying among STEMI patients those with the worst left ventricular morphological and functional indexes at follow-up.

Certainly, in the context of big complex outputs, several extrinsic and intrinsic methodological pitfalls should be inspected. However, upon the successful mitigation of foreseeable biases, the power of AI in cardiology, as well as in all medical specialties, will be tangible and pivotal, especially in the era of real world evidence (RWE).
